# MiR-106b inhibitors sensitize TRAIL-induced apoptosis in hepatocellular carcinoma through increase of death receptor 4

**DOI:** 10.18632/oncotarget.16707

**Published:** 2017-03-30

**Authors:** Changlong Xu, Liang Shi, Weilai Chen, Peipei Fang, Jie Li, Lingxiang Jin, Zhenzhen Pan, Chenwei Pan

**Affiliations:** ^1^ Department of Gastroenterology, The Second Affiliated Hospital and Yuying Children's Hospital of Wenzhou Medical University, Wenzhou 325027, China; ^2^ Department of Laboratory Medicine, The First Affiliated Hospital of Wenzhou Medical University, Wenzhou 325000, China; ^3^ Department of Neurology, Wenzhou People's Hospital, Wenzhou 325027, China; ^4^ Department of Infectious Disease, The Second Affiliated Hospital and Yuying Children's Hospital of Wenzhou Medical University, Wenzhou 325027, China; ^5^ Department of Infectious Disease, The First Affiliated Hospital of Wenzhou Medical University, Wenzhou 325000, China

**Keywords:** anti-miR-106b, TRAIL, DR4, HCC, apoptosis

## Abstract

TNF-related apoptosis-inducing ligand (TRAIL), which is a member of the TNF superfamily, can induce tumor cell apoptosis. However, multiple types of tumor, including hepatocellular carcinoma, show tolerance to TRAIL. Previous studies have demonstrated that tumor cells usually change their expression profile of microRNA (miRNA) to obtain the ability of tolerance to drugs. However, whether such change of miRNA on TRAIL sensitivity is seen in hepatocellular carcinoma still needs to be explored. In this study, we observed overexpression of miR-106b in both HCC patients’ tumor tissues and cell lines. Furthermore, we found that overexpression of miR-106b is associated with the sensitivity of TRAIL to HCC. Silencing of miR-106b with antisense oligonucleotide (anti-miR-106b) is proved to enhance the TRAIL-induced apoptosis and reduce the acquired drug resistance to TRAIL in HCC. Mechanically, we didn't observe the obvious change of pro-apoptotic proteins (Bax and Bid) and anti-apoptotic proteins (Bcl-2, Mcl-1 and Bcl-xl) after treatment of anti-miR-106b. However, we used the methods of bioinformatics, flow cytometry, cellular and molecular methods to prove that miR-106b directly targeted to death receptor 4 (DR4) 3′-UTR (3′-Untranslated Regions). MiR-106b inhibitors induced increase of DR4 expression and therefore enhancing TRAIL-mediated apoptosis in HCC. In summary, these results suggest the application of miR-106b inhibitors in HCC treatment. Combination with miR-106b inhibitors and TRAIL may be a novel clinical treatment method on HCC treatment in the future.

## INTRODUCTION

Hepatocellular carcinoma (HCC) is one of the leading causes of cancer deaths worldwide due to the late diagnosis and poor prognosis [1]. Although the surgery is the most effective treatment for early stage HCC patients, the cancer is unresectable for the patients with advanced HCC. Therefore, systemic chemotherapy and immunotherapy are considered as the alternative option [2, 3]. Unfortunately, despite advances in treatment strategies, the rate of 5-year survival remains very low, because HCC exhibits poor response to various kinds of treatments [4, 5]. Given this, it's urgent to explore novel and efficient approaches to raise the response of HCC cells to drugs.

TNF-related apoptosis-inducing ligand (TRAIL) is a member of the TNF superfamily. In TRAIL pathway, death receptors 4/5 (DR4/5) is able to bind with it to form the death-inducing signaling complex (DISC) followed by activating caspase-8-dependent apoptosis [6–8]. Previous reports have demonstrated that multiple types of cancer show high sensitivity to TRAIL-induced apoptosis both *in vitro* and *in vivo*. In addition, TRAIL treatment is harmless to normal cells. Thus, TRAIL has been tested as a promising new candidate for use in the treatment of cancer [9–11]. However, some cancer cells, including HCC, show tolerance to TRAIL-induced apoptosis [12]. Therefore, increasing the sensitivity of TRAIL will be of great help to explore the clinical application of it in cancer therapy.

Recent researches have emphasized close connection between microRNAs (miRNAs) and cancers. miRNAs are a class of endogenous, small, and non-coding RNAs that suppress the expression of target genes by binding to the complementary sequences of target mRNA 3′ UTR at the seed site [13, 14]. As miRNAs regulate numerous genes in cancer cells, they participate in a wide array of biological processes such as cell proliferation, differentiation, metabolism and apoptosis [15, 16]. In HCC, studies have demonstrated that miRNAs are usually dysregulated. Furthermore, ectopic expression of miRNAs induces chemoresistance, which causes low response of HCC cells to anti-tumor drugs [17–19]. In this study, we aim to investigate the relationship between miR-106b and sensitivity of HCC cells to TRAIL.

## RESULTS

### Expression of miR-106b is increased in HCC tissues and cell lines

**Figure 1:**
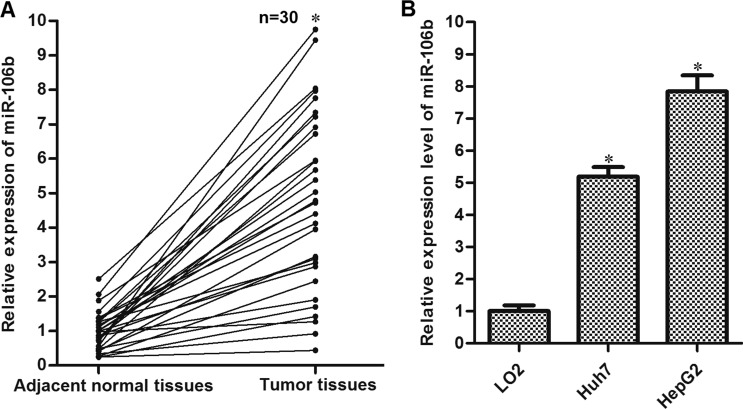
Overexpression of miR-106b in HCC (**A**) Tumor tissues and the adjacent normal tissues were obtained from HCC patients. QRT-PCR experiments were performed to analyze the expression of miR-106b in these samples. *P < 0.05 vs. adjacent normal tissues. (**B**) QRT-PCR experiments were performed to analyze the expression of miR-106b in LO2, Huh7 and HepG2. *P < 0.05 vs. LO2.

To explore the potential role of miR-106b in HCC, we detected the expression of miR-106b in thirty HCC patients’ samples. As shown in Figure 1A, expression of miR-106b was significantly increased in HCC tissues compared to their adjacent normal tissues. Furthermore, we observed that both the cultured HCC cell lines Huh7 and HepG2 expressed higher levels of miR-106b compared to the normal embryo liver cell line LO2 (Figure 1B). These results indicated that miR-106b may be a potential promoter in HCC.

### MiR-106b inhibitors enhance the anti-tumor effect of TRAIL in HCC cell lines

**Figure 2:**
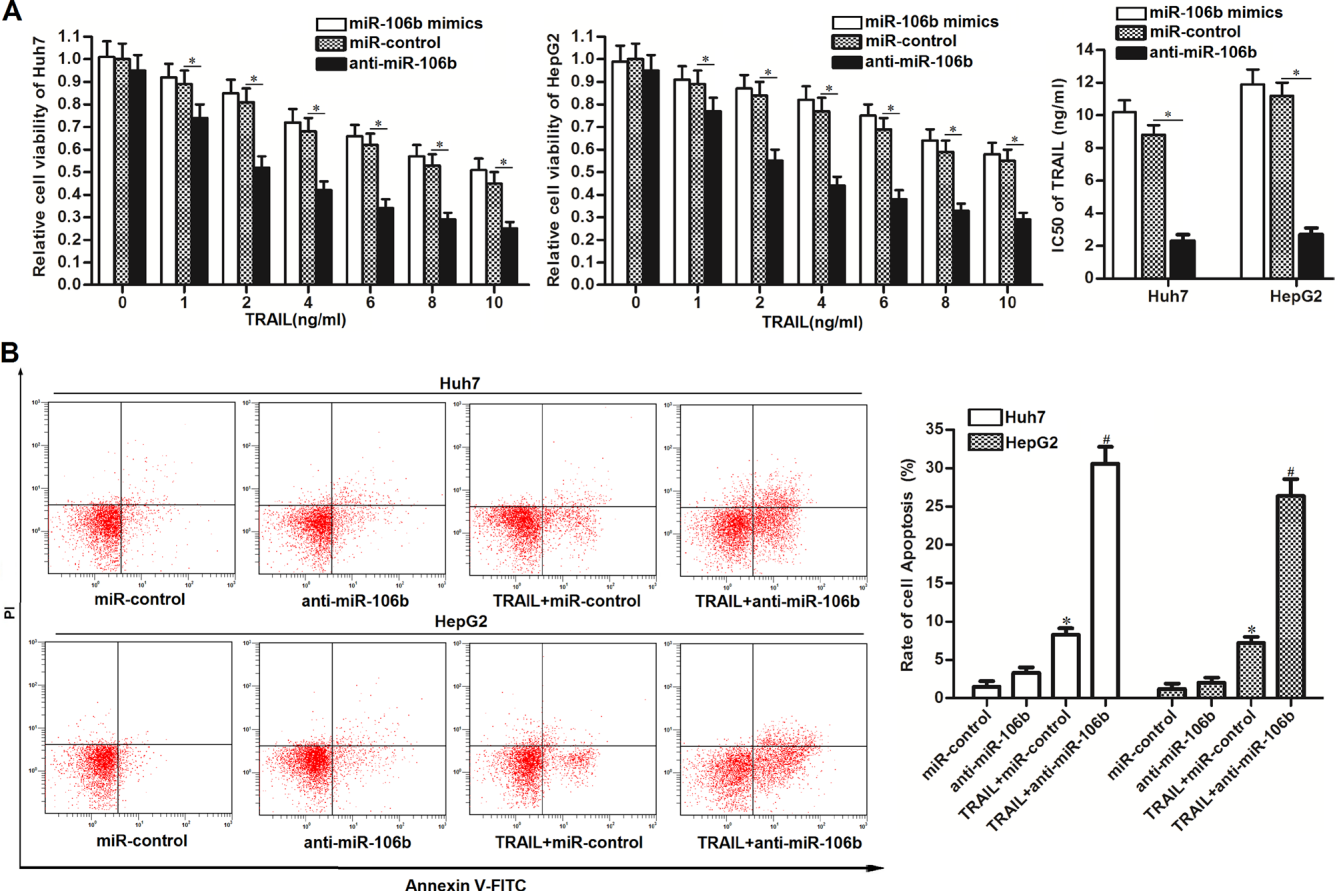
Anti-miR-106b enhances the TRAIL-induced apoptosis in HCC (**A**) CCK-8 cell viability assays were performed to evaluate the effect of miR-106b mimics and inhibitors on TRAIL-induced cell death. *P < 0.05. (**B**) After Huh7 and HepG2 cells were treated with TRAIL (2 ng/ml) and anti-miR-106b, flow cytometry analysis was performed to detect the cell apoptosis. *P < 0.05 vs. miR-control group. ^#^*P* < 0.05 vs. TRAIL + miR-106b group.

To explore the potential role of miR-106b in TRAIL-sensitivity to HCC, we performed CCK-8 cell viability assays and gain-and-loss experiments of miR-106b. We found that miR-106b mimics increased the cytotoxicity of TRAIL to HCC cell lines slightly. However, we observed that the miR-106b inhibitors (anti-miR-106b) dramatically enhanced the TRAIL-induced cell death of Huh7 and HepG2 cells. IC50 (half maximal inhibitory concentration) of TRAIL to miR-control transfected Huh7 and HepG2 cells was 1.82 and 2.14 fold higher than the anti-miR-106b transfected Huh7 and HepG2 cells, respectively (Figure 2A). As the TRAIL functions as an anti-tumor drug by inducing apoptosis in cancer cells, we next investigated the role of anti-miR-106b in TRAIL-induced apoptosis in Huh7 and HepG2. As expected, more apoptotic cells were observed in the group that treated with the combination with anti-miR-106b and TRAIL rather than the TRAIL single treatment group (Figure 2B). We therefore demonstrated that miR-106b inhibitors are able to enhance the anti-tumor effect of TRAIL on HCC through the apoptotic pathway.

### MiR-106b inhibitors increase the expression of DR4 in HCC cell lines

**Figure 3:**
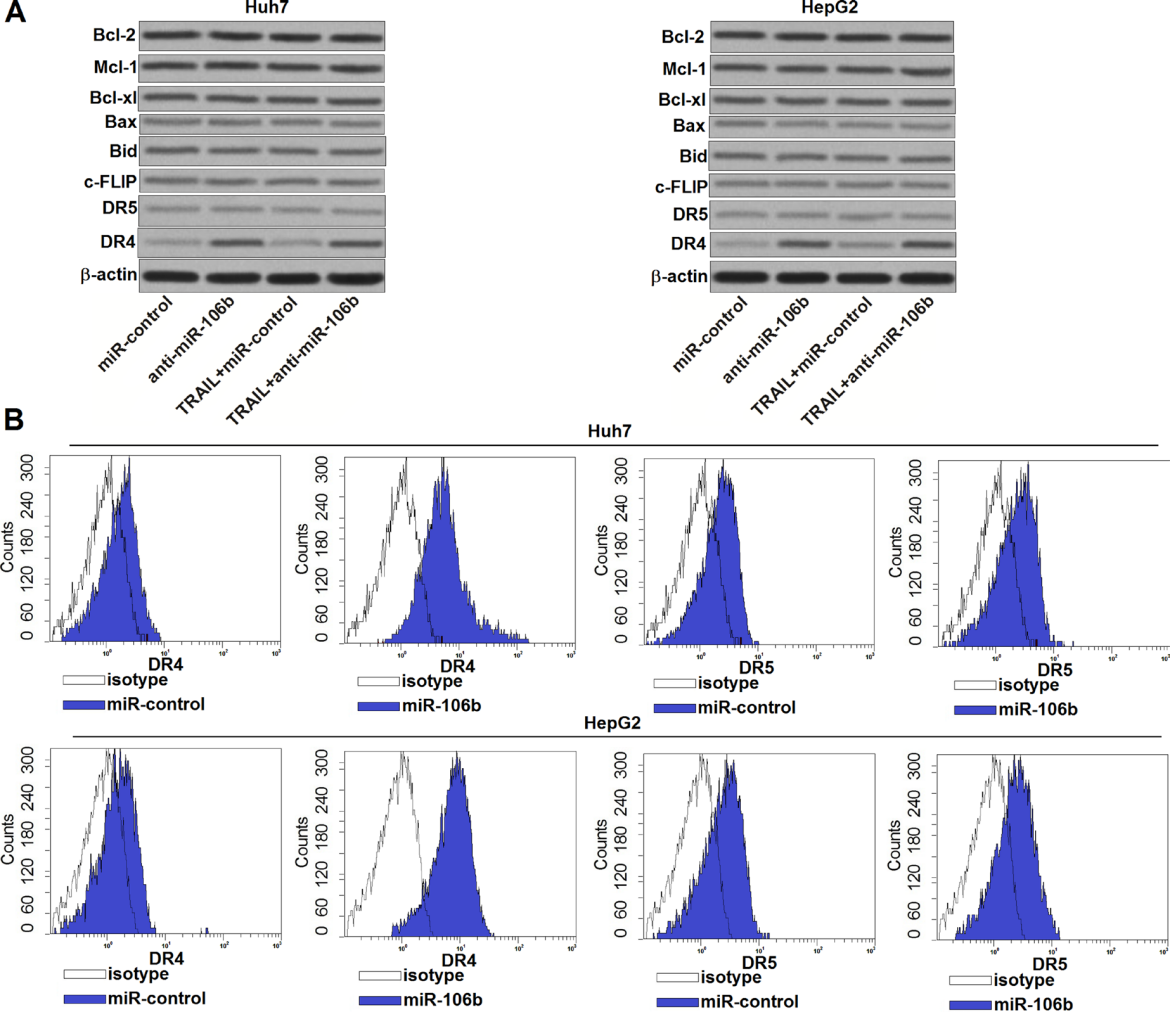
Anti-miR-106b increases the number of DR4 on the surface of HCC cells (**A**) After treatment with TRAIL (2 ng/ml) and anti-miR-106b (50 pmol/ml), western blot assays were performed to evaluate the expression of Bcl-2 family proteins and DR4/5 in Huh7 and HepG2. (**B**) After transfection with anti-miR-106b, flow cytometry analysis was performed to detect the number of DR4/5 on the cell surface of Huh7 and HepG2.

To explore the potential mechanisms by which anti-miR-106b increases the sensitivity of HCC cells to TRAIL, we performed western blot assays to detect the expression of c-FLIP and Bcl-2 family proteins which are master regulators of cell survival and apoptosis [20]. However, transfection with anti-miR-106b didn't induce obvious change of pro-apoptotic proteins (Bax and Bid) and anti-apoptotic proteins (Bcl-2, Mcl-1, Bcl-xl and c-FLIP) (Figure 3A). Since TRAIL signaling induces apoptosis by binding to DR4 and DR5 [21], we next investigated whether miR-106b inhibitors changed the expression of DR4/5. We found that the expression of DR4 but not the DR5 was significantly increased due to the anti-miR-106b treatment (Figure 3A). Furthermore, the results of flow cytometry analysis showed that anti-miR-106b obviously increased the number of DR4 on the surface of Huh7 and HepG2, but didn't influence the DR5 (Figure 3B). These results demonstrated that miR-106b inhibitors have the ability to increase the number of DR4 to enhance the TRAIL pathway in HCC.

### DR4 is the target of miR-106b in HCC

**Figure 4:**
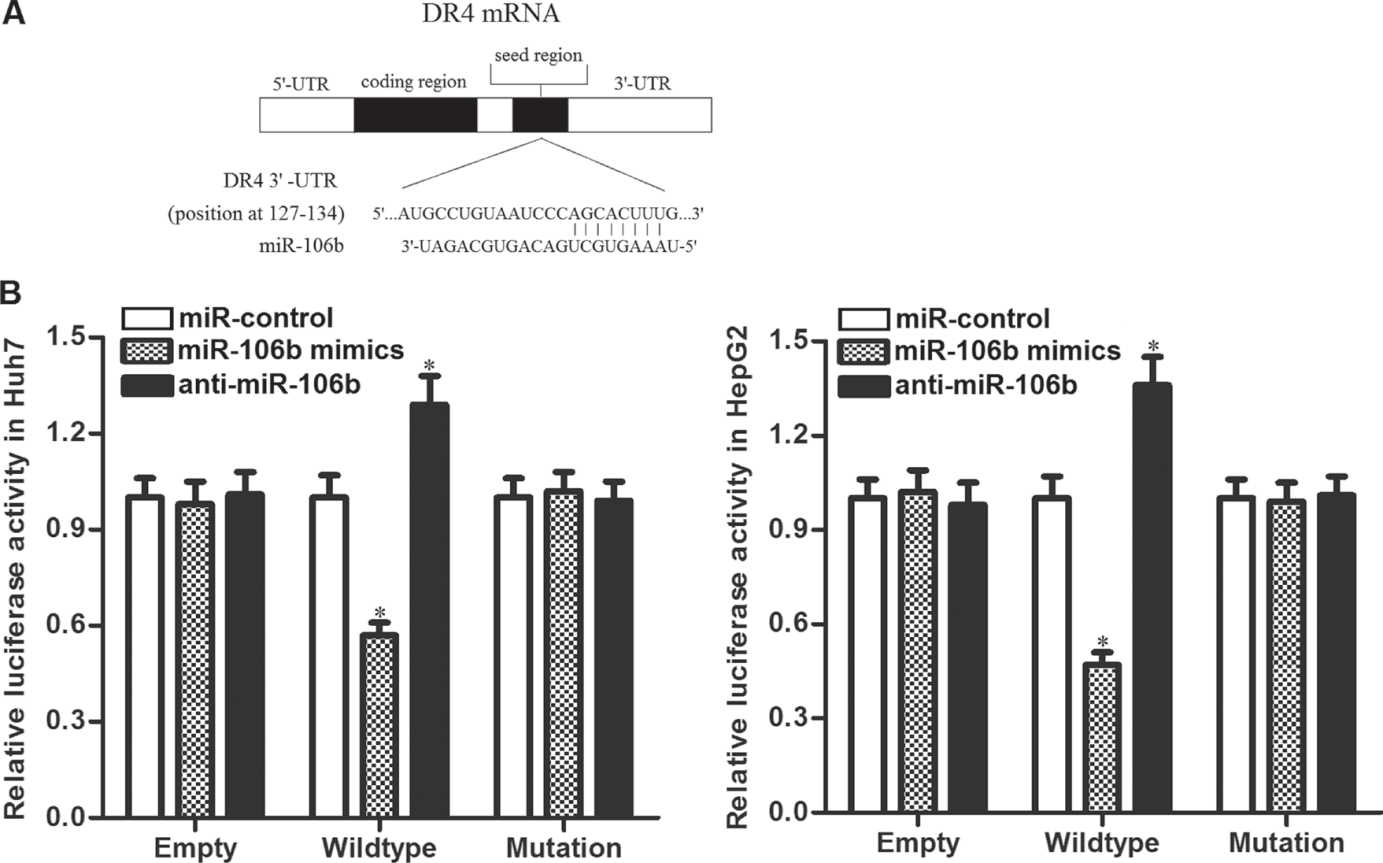
DR4 is the direct target of miR-106b (**A**) Complementary pairing site between miR-106b and DR4 3′ UTR showed by TargetScan database. (**B**) Huh7 and HepG2 cells were co-transfected with miR-106b mimics (or miR-106b inhibitors) and luciferase reporters contained wildtype/mutant 3′-UTR of DR4. 48 h post transfection, luciferase activity was detected using Dual-Luciferase Reporter Assay System according to the manufacturer's instruction. *P < 0.05 vs. miR-control group.

Preceding results indicated that miR-106b inhibitors increased the DR4 expression. So we tried to explore the molecular mechanisms responsible for the function of anti-miR-106b that was observed above. After searching the potential targets of miR-106b on the public miRNA database of TargetScan, we found that the 3′ UTR of DR4 mRNA contained complementary pairing site at the position of 127–134 (Figure 4A). To confirm that miR-106 b directly interacts with DR4 3′ UTR, we cloned the corresponding 3′-UTR fragment of DR4 into the pMIR reporters and subsequently performed the luciferase assays. As shown in Figure 4B, transfection with miR-106b mimics significantly decreased the luciferase activities of the pMIR reporters contained wildtype but not the mutant 3′-UTR of DR4. In contrast, anti-miR-106b significantly increased the luciferase activities of the wildtype pMIR reporters. These results proved that DR4 is the target of miR-106b in HCC.

### MiR-106b inhibitors enhance the TRAIL signaling by increasing the expression of DR4 in HCC cell lines

**Figure 5:**
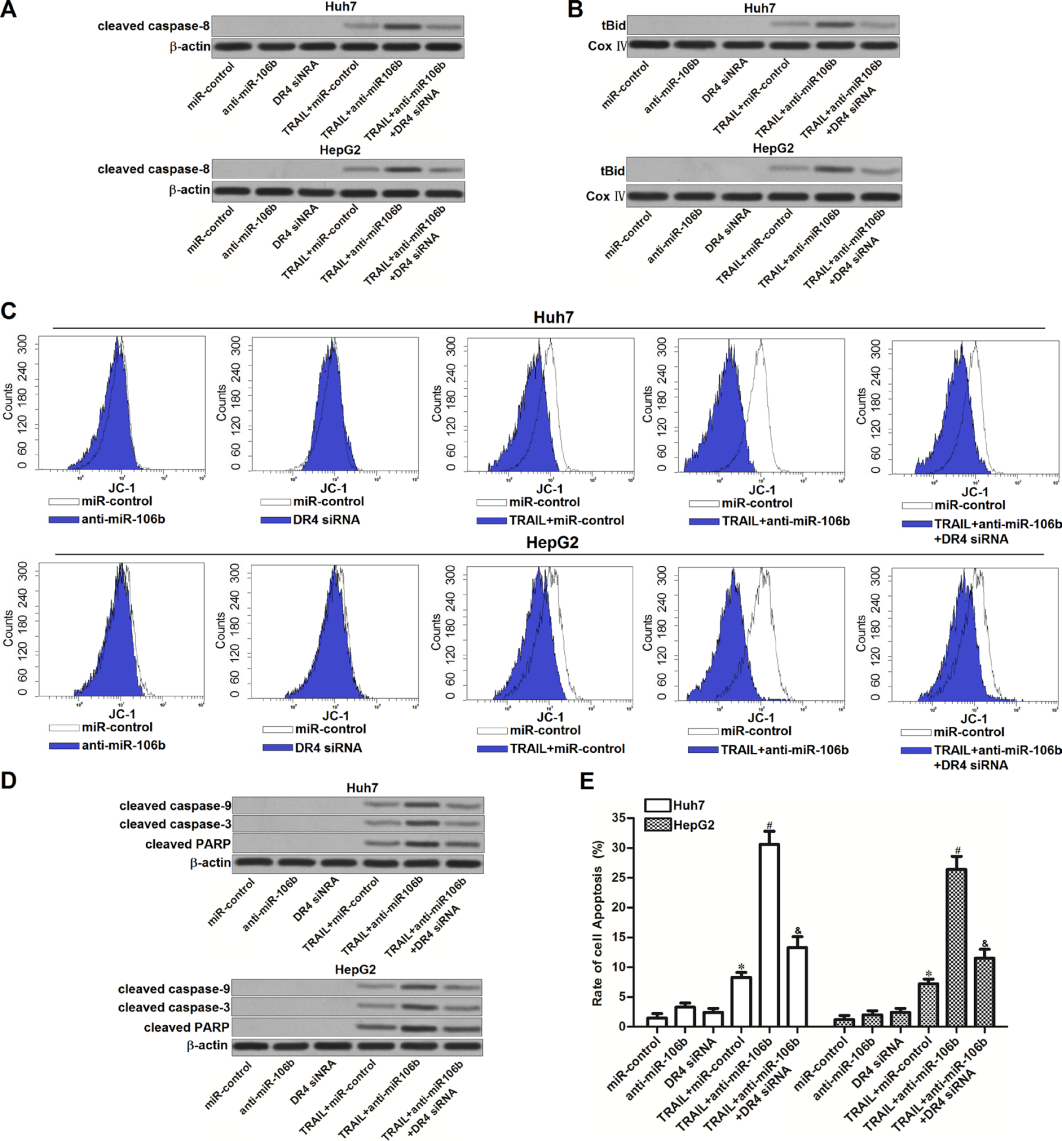
Effect of miR-106b inhibitors on TRAIL-dependent apoptotic pathway (**A**) After treatment with TRAIL (2 ng/ml), anti-miR-106b (50 pmol/ml) and DR4 siRNA (50 pmol/ml), western blot analysis was performed to evaluate the effect of anti-miR-106b on TRAIL-dependent activation of caspase-8. (**B**) After treatment with TRAIL (2 ng/ml), anti-miR-106b (50 pmol/ml) and DR4 siRNA (50 pmol/ml), mitochondria were seperated from Huh7 and HepG2 cells, expression level of tBid was evaluated by western blot analysis. Cox IV was detected as internal control of mitochondrial proteins. (**C**) After treatment with TRAIL (2 ng/ml), anti-miR-106b (50 pmol/ml) and DR4 siRNA (50 pmol/ml), mitochondrial membrane potential (MMP) of Huh7 and HepG2 cells was detected by flow cytometry by using JC-1 staining. (**D**) After treatment with TRAIL (2 ng/ml), anti-miR-106b (50 pmol/ml) and DR4 siRNA (50 pmol/ml), cleavage of caspase-9, caspase-3 and PARP in TRAIL signaling pathway was detected by western blot analysis. (**E**) After treatment with TRAIL (2 ng/ml), anti-miR-106b (50 pmol/ml) and DR4 siRNA (50 pmol/ml), cell apoptosis of Huh7 and HepG2 was measured after they were treated with TRAIL and RNA oligoribonucleotides.

Our preceding results have showed that anti-miR-106b sensitized Huh7 and HepG2 cells to TRAIL-induced apoptosis. We next investigated the role of anti-miR-106b in TRAIL signaling pathway. As TRAIL-DR4 signaling initiates caspase-8-dependent apoptosis (8), we first detected the activation of caspase-8 in HCC cells treated with anti-miR-106b and TRAIL. As shown in Figure 5A, anti-miR-106b significantly enhanced the activation of caspase-8 induced by TRAIL. In addition, knockdown of DR4 by specific siRNA was observed to abolish the effect of anti-miR-106b. It indicated that anti-miR-106b promoted activation of caspase-8 is dependent on the increase of DR4. Bid is the substrate of caspase-8, which can be cleaved by caspase-8 as tBid and translocated to mitochondria (22). As expected, we found that anti-miR-106b was able to promote the translocation of tBid to mitochondria in TRAIL pathway (Figure 5B). Researches have demonstrated that accumulation of tBid on mitochondria membranes induces mitochondrial apoptosis cells (23). According to our results of flow cytometry, we found that combination with anti-miR-106b and TRAIL induced significant decrease of mitochondrial membrane potential (MMP), despite DR4 siRNA inhibited the collapse of mitochondria (Figure 5C). It's indicated that miR-106b inhibitors are able to enhance the mitochondrial apoptosis induced by TRAIL signaling through upregulating the expression of DR4. As the results, caspase-9 and caspase-3 was activated, and PARP which is the substrate of caspase-3 was cleaved (Figure 5D). In the final step, combination with anti-miR-106a and TRAIL induced significant apoptosis in Huh7 and HepG2 cells (Figure 5E). Taken together, we demonstrated that miR-106b inhibitors enhance the TRAIL-dependent apoptosis by increasing the expression of DR4 in HCC cell lines.

### MiR-106b inhibitors sensitize HCC cells to TRAIL *in vivo*

**Figure 6:**
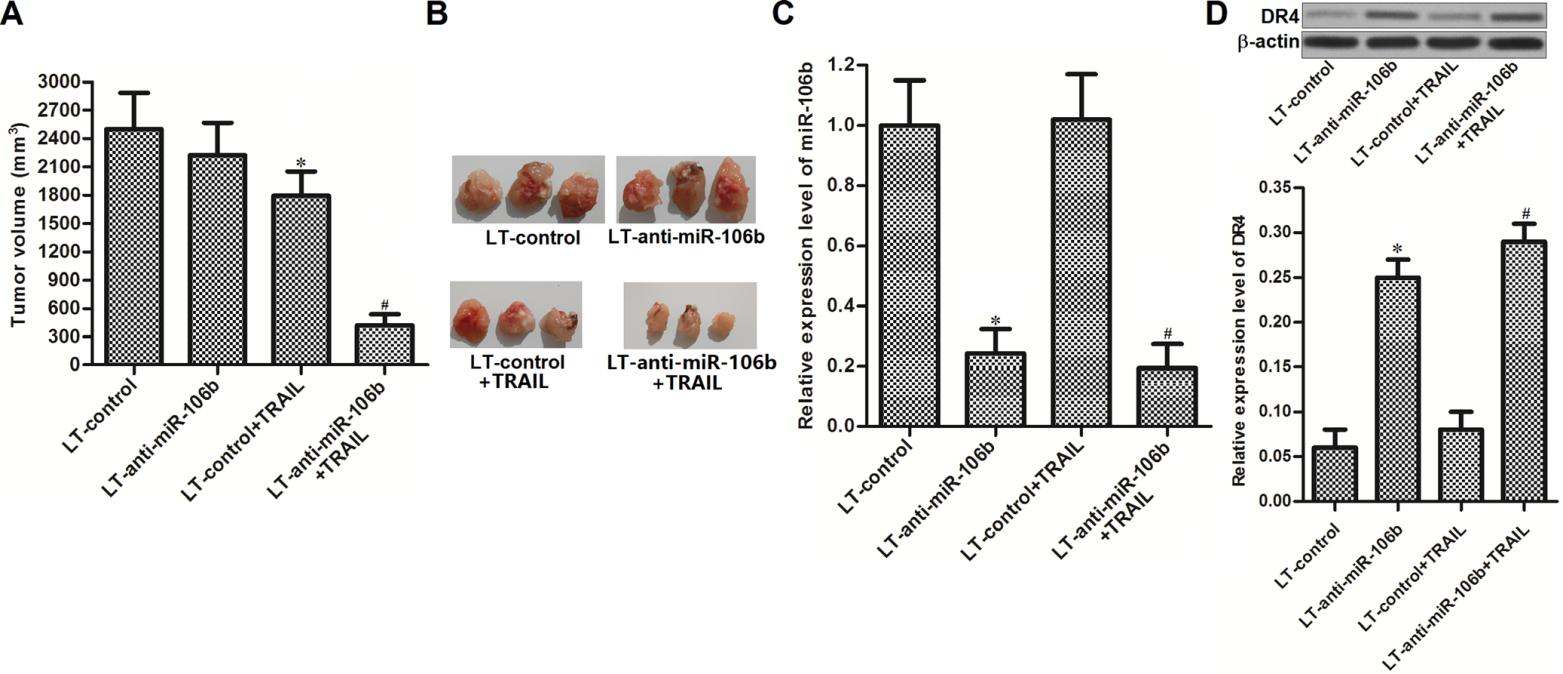
MiR-106b inhibitors sensitize HCC cells to TRAIL *in vivo* (**A**) Tumor volumes of lentivirus transfected HepG2 *in vivo* model were assessed on day 28 post-injection. (**B**) Tumor tissues obtained from the HepG2 *in vivo* model. (**C**) Expression level of miR-106b in HepG2 tumor tissues was detected by qRT-PCR analysis. *P < 0.05 vs. LT-control group. ^#^*P* < 0.05 vs. LT-control + TRAIL group. (**D**) Expression level of DR4 in HepG2 tumor tissues was detected by western blot analysis.*P < 0.05 vs. LT-control group. ^#^*P* < 0.05 vs. LT-control + TRAIL group.

To investigate whether miR-106b inhibitors increased the anti-tumor effect of TRAIL *in vivo*, we established the *in vivo* model of HCC by using lentivirus transfected HepG2 cells (LT-control transfected HepG2 or LT-anti-miR-106b transfected HepG2). We didn't observe obvious difference of tumor sizes between LT-control group and LT-anti-miR-106b group. However, inhibition of miR-106b significantly enhanced the anti-tumor effect of TRAIL in HCC *in vivo* model (Figure 6A and 6B). After analyzing the expression of miR-106b and DR4 in tumor tissues derived from HepG2 *in vivo* model, we observed low expression level of miR-106b and high expression level of DR4 in LT-anti-miR-106b transfected HepG2 cells (Figure 6C and 6D). These results demonstrated that miR-106b inhibitors enhanced the anti-tumor effect of TRAIL by increasing the expression of DR4 on HCC *in vivo* model.

### MiR-106b inhibitors reverse the drug-resistance of HCC to TRAIL

**Figure 7:**
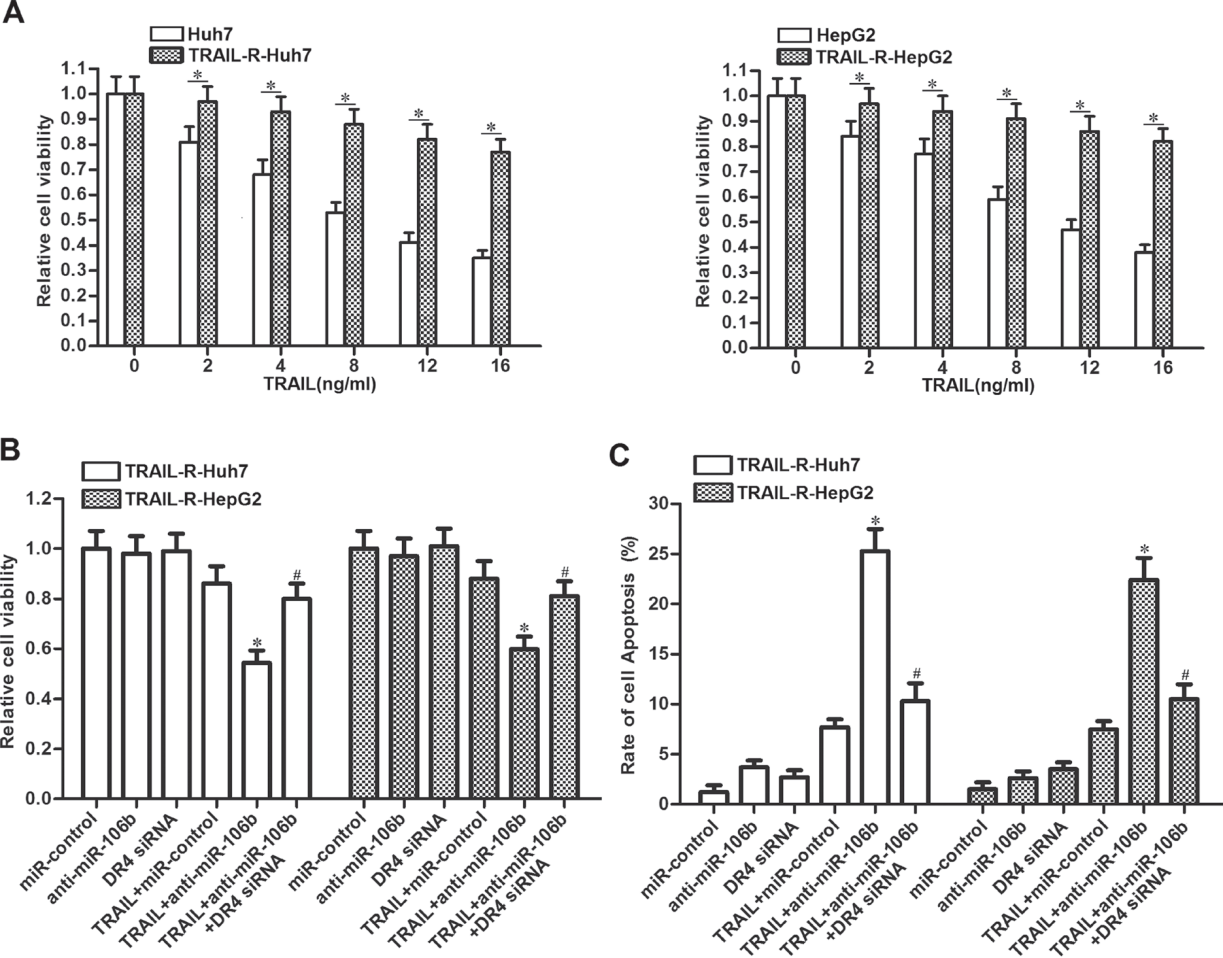
Anti-miR-106b reverses drug-resistance of HCC to TRAIL (**A**) Response of Huh7, TRAIL-R-Huh7, HepG2 and TRAIL-R-HepG2 cells to TRAIL was evaluated by CCK-8 cell viability assays. *P < 0.05. (**B**) After treatment with TRAIL (10 ng/ml), anti-miR-106b (50 pmol/ml) and DR4 siRNA (50 pmol/ml), cell viability of TRAIL-R-Huh7 and TRAIL-R-HepG2 was detected by CCK-8 assays. *P < 0.05 vs. TRAIL + miR-control group. ^#^*P* < 0.05 vs. TRAIL + anti-miR-106b group. (**C**) After treatment with TRAIL (10 ng/ml), anti-miR-106b (50 pmol/ml) and DR4 siRNA (50 pmol/ml), cell apoptosis of TRAIL-R-Huh7 and TRAIL-R-HepG2 was detected by flow cytometry. *P < 0.05 vs. TRAIL + miR-control group. ^#^*P* < 0.05 vs. TRAIL + anti-miR-106b group.

TRAIL has yet to be successfully used in clinical predominantly because of the rapid onset of TRAIL resistance (24). We therefore established the TRAIL-resistant Huh7 and HepG2 models to investigate the relationship between miR-106b inhibitors and TRAIL-resistance in HCC. As shown in Figure 7A, TRAIL resistant Huh7 (TRAIL-R-Huh7) and TRAIL resistant HepG2 (TRAIL-R-HepG2) exhibited significant low response to TRAIL treatment. However, we observed that transfection with anti-miR-106b obviously increased the cytotoxicity of TRAIL to TRAIL-R-Huh7 and TRAIL-R-HepG2 via suppressing the expression of DR4 (Figure 7B). In addition, results of flow cytometry showed that transfection with anti-miR-106b was able to increase the apoptosis of TRAIL-R-Huh7 and TRAIL-R-HepG2 cells which were treated with TRAIL (Figure 7C). These results demonstrated that MiR-106b inhibitors are able to reverse the drug-resistance of HCC to TRAIL.

## DISCUSSION

Accumulating evidences have demonstrated that aberrant expression of miRNAs induces drug-resistance in cancers, and lead to low response to cancer therapy [25, 26]. Among these dysregulated miRNAs, miR-106b is reported to promote cancer development, proliferation and metastasis in multiple tumors including HCC. It has been reported that overexpression of miR-106b activates the epithelial-mesenchymal transition (EMT) process of HCC to promote cell migration and metastasis [27]. Knockdown of miR-106b resulted in reduction of cell proliferation in renal cell carcinoma and HCC [28, 29]. These studies indicate that miR-106b acts as a potential oncogene.

TRAIL is an anti-tumor cytokine, which selectively kills cancer cells through apoptotic pathway without damaging normal cells. However, low sensitivity to TRAIL becomes a big obstacle for the use of it in some cancer types [30]. In the present study, we observed the overexpression of miR-106b in HCC. Moreover, our results demonstrated that miR-106b was associated with sensitivity of HCC cells to TRAIL. Knockdown of miR-106b by its antisense oligodeoxynucleotides was able to elevate the response of HCC cells to the apoptosis induced by the TRAIL signaling.

In TRAIL signaling pathway, cleavage of caspase-8 is the incidence followed by the coupling of the TRAIL with its receptor of DR4 and DR5. As the results of caspase-8 activation, Bid, which is one pro-apoptotic protein, is subsequently triggered as truncated Bid (tBid) [31, 32]. tBid targets to mitochondrial outer membrane to induce the collapse of mitochondrial membrane potential (MMP) in the presence of Bax and Bak to induce the mitochondria apoptosis [33, 34]. In this study, we aim to explore the mechanisms by which miR-106b inhibitors increased the cytotoxicity of TRAIL to HCC. Our data showed that miR-106b inhibitors didn't change the expression of Bcl-2 family proteins but increase the amount of DR4 on the HCC cell surface. Indeed, the TRAIL signaling was augmented due to increase of DR4 expression. Subsequently, we found that miR-106b inhibitors enhanced the cleavage of caspase-8, -9, -3 and mitochondrial apoptosis which was TRAIL-dependent. In general, our evidences demonstrate that miR-106b inhibitors have the ability to strengthen the TRAIL apoptotic signaling through increasing the expression of DR4.

Previous studies have pointed out that rapid tolerance of TRAIL often happens in cancer therapy, and it is a problem needs to be solved urgently [35, 36]. In the present study, we established the TRAIL-resistant HCC models to study the role of miR-106b in the acquired resistance to TRAIL. Interestingly, our results demonstrate that miR-106b inhibitors are able to weaken the TRAIL-resistance and increase the cytotoxicity of TRAIL in HCC models.

In summary, this study suggests the application of miR-106b inhibitors in TRAIL treatment. Combination with miR-106b inhibitors may be a novel strategy to overcome the rapid tolerance of TRAIL in HCC treatment in the future.

## MATERIALS AND METHODS

### Cell lines and tissue samples

Human HCC cell lines (Huh7 and HepG2) and human normal embryo liver cell line (LO2) were obtained from the Institute of Biochemistry and Cell Biology, Chinese Academy of Sciences (Shanghai, China). Cell lines were cultured in DMEM basic medium (Gibco, USA) with 10% fetal bovine serum (FBS, Gibco, USA) at 37°C in a humidified incubator with 5% CO_2_. A total of 30 primary hepatocellular carcinoma tissues and their adjacent normal tissues were obtained from patients who underwent tumor resection in The Second Affiliated Hospital & Yuying Children's Hospital of Wenzhou Medical University from 5/2014 to 11/2016. Use of tumor samples in the present study was approved by the ethics committee of The Second Affiliated Hospital & Yuying Children's Hospital of Wenzhou Medical University. All of the patients had given their informed consent.

### Quantitative real-time polymerase chain reaction (qRT-PCR) for miR-106b expression

Total RNAs in tissues and cell lines were extracted by using TRIzol reagent (Invitrogen, USA). For amplification of miR-106b, reverse transcription experiment was performed by PrimeScript RT reagent Kit (TaKaRa, Japan). Subsequently, real-time PCR experiments were performed by using the SYBR Premix Ex Taq (TaKaRa) on an ABI PRISM 7900 Sequence Detection System (Applied Biosystems, USA) according to the manufacturer's protocol. Quantization of U6 snRNA was used to normalize the expression level of miR-106b.

### RNA oligoribonucleotides and transfection

All of the RNA oligoribonucleotides [miR-106b mimics, miR-106b inhibitor (anti-miR-106b), miR-control and DR4 siRNA] were purchased from Genechem Co., Ltd (Shanghai, China). Anti-miR-106b is 2′-O-methyl-modified RNA oligoribonucleotides with the sequences complementary to the mature miR-106b. The negative control RNA duplex (miR-control) for miR-106b mimic, anti-miR-106b and the DR4 siRNA was nonhomologous to any human genome sequences. For transfection, RNA oligoribonucleotides (50 pmol/ml) were transiently transfected into the HCC cells with Lipofectamine 2000 reagent (Invitrogen, USA) according to the manufacturer's instructions.

### Measurement of cell viability

Huh7 and HepG2 cells were seeded in 96-well plates overnight at the density of 5 × 10^3^ per well. Cells were transfected with RNA oligoribonucleotides before treatment with different concentrations of TRAIL (R&D system, USA). After incubation with TRAIL for 48 h, cell viability was measured by using CCK-8 detection kit (Sigma-Aldrich, USA) according to the manufacturer's protocol. IC50 (half maximal inhibitory concentration) of TRAIL to Huh7 and HepG2 cells was calculated acoording to the results of CCK-8 cell viability assays.

### Luciferase assays

Human DR4 mRNA 3′ UTR sequences were amplified and inserted into pMIR Firefly luciferase reporters (Ambion, USA). Reporters contained mutant DR4 mRNA 3′ UTR were created by using the site-directed mutagenesis kit (Takara, Japan). Firefly luciferase reporters, Renilla luciferase pRL-TK vector (used as internal control, Promega, USA) and miR-106b mimics (or anti-miR-106b) were co-transfected into the Huh7 and HepG2 cells. For luciferase assays, cells were lysed and then the Firefly and Renilla luciferase activities were detected by using Dual-Luciferase Reporter System Kit (Promega) according to the manufacturer's protocol.

### Separation of mitochondria and western blot analysis

To separate mitochondria from cytoplasm in Huh7 and HepG2 cells, mitochondria/Cytosol Fraction Kit (BioVision, USA) was used according to the manufacturer's protocol. Mitochondria -derived proteins or total proteins in Huh7 and HepG2 were extracted by using radio immunoprecipitation assay (RIPA) lysis buffer (Cell Signaling, USA). Concentrations of each protein sample were determined by BCA Assay Kit (Pierce, USA). Equal amounts of proteins in each sample were separated by 12.5% sodium dodecyl sulfate-polyacrylamide gel electrophoresis (SDS-PAGE) and transferred to polyvinylidene fluoride (PVDF) membranes (Millipore, Billerica, MA, USA). Membranes were then probed with primary antibodies (anti-Bcl-2, anti-Mcl-1, anti-Bcl-xl, anti-Bax, anti-Bid, anti-c-FLIP, anti-DR5, anti-DR4, anti-cleaved caspase-8, anti-cleaved caspase-9, anti-cleaved caspase-3, anti-cleaved PARP, anti-Cox IV and anti-β-actin, Cell Signaling) overnight. Subsequently, the Membranes were incubated with suitable horseradish peroxidase-conjugated secondary antibodies for 2 h. Blots were visualized by using enhanced chemilu-minescence detection kit (Pierce).

### Flow cytometry analysis

For detecting the expression level of DR4 and DR5 in cell surface of Huh7 and HepG2, cells were incubated with anti-DR4-PE or anti-DR5-PE (R&D System) followed by analyzing on a flow cytometry (Becton Dickinson, USA). For measurement of cell apoptosis, Huh7 and HepG2 cells were incubated with Annexin V/Propidium Iodide (Sigma-Aldrich) and analyzed by using flow cytometry according to the manufacturer's instructions. For measurement of mitochondrial membrane potential (MMP), Huh7 and HepG2 cells were incubated with 5 μM JC-1 (Molecular Probes, USA) followed by FACS analysis.

### Establishment of TRAIL-resistant Huh7 and HepG2 models

TRAIL resistant Huh7 (TRAIL-R-Huh7) and TRAIL resistant HepG2 (TRAIL-R-HepG2) models were established by continuous exposure to TRAIL as previously described [8]. Before the experiments were performed, TRAIL-R-Huh7 and TRAIL-R-HepG2 cells were cultured in TRAIL-free medium for 2 weeks to eliminate the effect of remanent TRAIL.

### Animal models and treatment

Animal experiments were carried out in four-week-old female immunodefcient nude BALB/c mice were purchased from Shanghai Super-B&K Laboratory Animal Corp., Ltd. (Shanghai, China). For xenograft, HepG2 cells were transfected with 5 × 10^5^ transducing units of empty lentivirus (LT-control) or recombinant lentivirus contained miR-106b antisense nucleotide sequences (LT-anti-miR-106b) (Genechem Co., Ltd). 5 × 10^6^ transfected HepG2 were injected into animals to establish a subcutaneous tumor model. After 7 days, the animals were randomized into 4 treatment groups and received intraperitoneal (i.p.) injections of 40 μg/kg TRAIL per two days. Tumor volume was assessed every three days for a total of 28 days post-injection. The animal care and experimental protocols were approved by the Animal Care Committee of The Second Affiliated Hospital & Yuying Children's Hospital of Wenzhou Medical University.

### Statistical analysis

Data are represented as mean ± SD and analyzed by using SPSS 15.0 software. Student's t test and ANOVA analysis were used to compare mean values. A *P* < 0.05 was considered to be statistically significant. All the experiments were independently repeated at least 3 times.
